# Multiple myeloma cell lines and primary tumors proteoma: protein biosynthesis and immune system as potential therapeutic targets

**DOI:** 10.18632/genesandcancer.88

**Published:** 2015-11

**Authors:** Rodrigo Carlini Fernando, Fabricio de Carvalho, Diego Robles Mazzotti, Adriane Feijó Evangelista, Walter Moisés Tobias Braga, Maria de Lourdes Chauffaille, Adriana Franco Paes Leme, Gisele Wally Braga Colleoni

**Affiliations:** ^1^ Departamento de Oncologia Clínica e Experimental, Disciplina de Hematologia e Hemoterapia, Universidade Federal de São Paulo, UNIFESP, São Paulo, Brazil; ^2^ Departamento de Psicobiologia, Universidade Federal de São Paulo, UNIFESP, São Paulo, Brazil; ^3^ Fundação Pio XII - Hospital de Câncer de Barretos, Barretos, Brazil; ^4^ Laboratório de Espectrometria de Massas, Laboratório Nacional de Biociências, LNBio, Conselho Nacional de Pesquisa em Energia e Materiais, CNPEM, Campinas, Brazil

**Keywords:** multiple myeloma, proteomics, therapeutic targets

## Abstract

Despite great advance in multiple myeloma (MM) treatment since 2000s, it is still an incurable disease and novel therapies are welcome. Therefore, the purpose of this study was to explore MM plasma cells' (MM-PC) proteome, in comparison with their normal counterparts (derived from palatine tonsils of normal donors, ND-PC), in order to find potential therapeutic targets expressed on the surface of these cells. We also aimed to evaluate the proteome of MM cell lines with different genetic alterations, to confirm findings obtained with primary tumor cells. Bone marrow (BM) samples from eight new cases of MM and palatine tonsils from seven unmatched controls were submitted to PC separation and, in addition to two MM cell lines (U266, RPMI-8226), were submitted to protein extraction for mass spectrometry analyses. A total of 81 proteins were differentially expressed between MM-PC and ND-PC - 72 upregulated and nine downregulated; U266 vs. RPMI 8226 cell lines presented 61 differentially expressed proteins - 51 upregulated and 10 downregulated. On primary tumors, bioinformatics analyses highlighted upregulation of protein biosynthesis machinery, as well as downregulation of immune response components, such as MHC class I and II, and complement receptors. We also provided comprehensive information about U266 and RPMI-8226 cell lines' proteome and could confirm some patients' findings.

## INTRODUCTION

First described in the mid-1800s [1,2], multiple myeloma (MM) is a plasma cell (PC) malignancy, characterized by bone marrow (BM) clonal PC infiltration, presence of monoclonal protein in serum and/or urine and evidence of end-organ or tissue damage [3]. It is the second most prevalent hematologic malignancy [4], and American Cancer Society estimates that in 2015 about 26,850 new MM cases will be diagnosed and 11,240 disease related deaths will occur in the United States [5]. The median age at diagnosis is 69 years, and 61.8% of MM patients are 65 years or older at diagnosis [6].

A breakthrough in MM treatment has occurred since 2000s, with the introduction of immunomodulatory (thalidomide, lenalidomide, and pomalidomide) and proteasome inhibitor (bortezomib and carfizomib) drugs [7]. As a result, response rates, progression-free survival, overall survival and quality of life of patients have increased considerably [8]. However, some patients remain refractory to current anti-MM treatments, and even those who achieve a complete response after autologous stem-cell transplant will relapse at some point. Thus, MM is still an incurable disease and novel therapies are welcome.

Tumor microenvironment plays a key role in the maintenance and progression of several types of cancer [9], including solid tumors [10] and hematologic malignancies [11]. In MM, tumor microenvironment is represented by BM, where MM tumor cells interact with normal components, such as bone marrow stromal cells, osteoblasts, osteoclasts, endothelial cells, and extracellular matrix, promoting proliferation, migration, survival and drug resistance of tumor cells [12]. This interaction occurs directly and indirectly, through cell adhesion- and cytokine/growth factors-mediated mechanisms, respectively [12].

Proteomic studies seem to be a useful tool for a better understanding of MM development, as well as for the discovery of potential therapeutic targets and biomarkers [13]. However, there are a limited number of published studies on MM proteomics. Most of them evaluated only MM cell lines and, moreover, they provide fragmented information [13,14]. Therefore, this work can be helpful to fill the gap in the literature about this subject, since surface proteins are attractive targets in MM treatment, such as CD38 and the respective monoclonal antibody daratumumab, which is being tested in clinical trials [15].

Therefore, the purpose of this study was to explore the proteome of MM cells, in comparison with their normal counterparts (derived from palatine tonsils of normal donors), in order to find novel potential therapeutic targets expressed on the surface of these cells, capable of reducing the relapse rate and increasing the quality of life of these patients. We also aimed to evaluate the proteome of MM cell lines with different genetic alterations, to confirm findings obtained with primary tumor cells

## RESULTS

### Subjects

All MM patients had advanced stage disease (Durie- Salmon stage II or III and/or ISS stage ≥ 2). The median age at diagnosis was 68.5 years (ranging from 38 to 80) and 75% of cases were male (Table 1).

**Table 1:** Baseline characteristics of MM patients*

Characteristics	Cases (N=8)
Age (years)	
Median	68.5
Range	38-80
Gender (%)	
Female	25.0
Male	75.0
Isotype (%)	
IgG	50
IgA	37.5
LC^1^	12.5
Light chain (%)	
λ	38.0
k	62.0
ISS^2^	
1	12.5
2	12.5
3	75.0

1 LC = Light Chain.

2 ISS = International Staging System.

* Controls (palatine tonsils) were not matched by age or gender.

### MM Cell Line Culture and Cytogenetic Analyses by FISH

**Figure 1:**
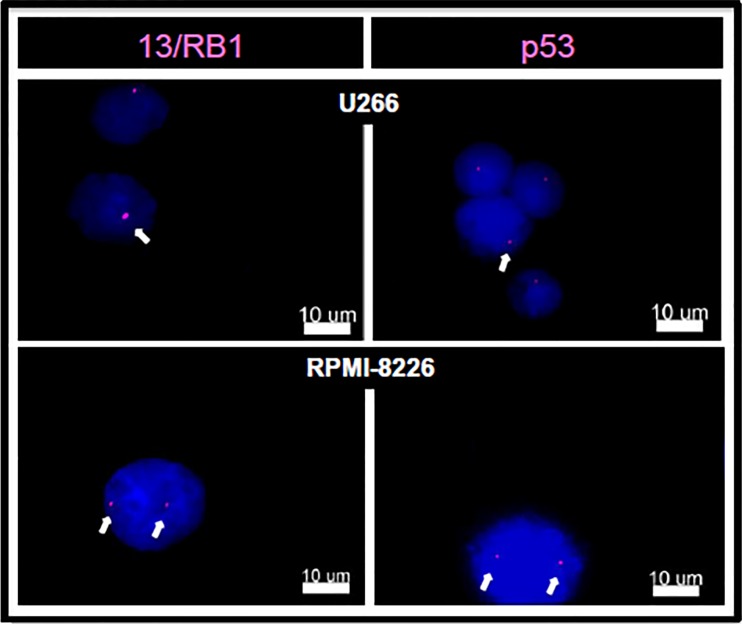
Cytogenetic analyses of MM cell lines (U266, RPMI-8226) by FISH technique We evaluated the deletion of chromosome 13 (13q14 LSI 13 [RB1] Spectrum Orange, Vysis) and deletion of 17p (17p13.1 LSI p53 Spectrum Orange, Vysis). U226 showed deletions of 13 and 17p; RPMI-8226 has no abnormalities. The images were obtained under a microscope Olympus BX60, MacProbe v4.2.3 software. Increase 100X.

According to http://www.keatslab.org, U266 cell line has E419X missense mutation on *RB1* gene and A161T missense mutation on *TP53* gene, while RPMI-8226 cell line has E285K missense mutation on *TP53* gene. Although, we have not screened for these mutations in our MM cell lines, our U266 cell line has shown deletions of chromosome 13 and 17p, which involve, respectively, *RB1* and *TP53* genes, whereas RPMI-8226 cell line has shown neither deletion of chromosome 13, nor deletion of chromosome 17p (Figure 1).

### Protein Extraction/Quantification and Mass Spectrometry Analyses

Protein quantity obtained after extraction ranged from 52 μg to 126 μg. 30 μg of peptide solution from MM patients and normal donors' pools and 50 μg of peptide solution from each cell line was used for iTRAQ reagents labeling.

### Bioinformatics Analyses

**Figure 2:**
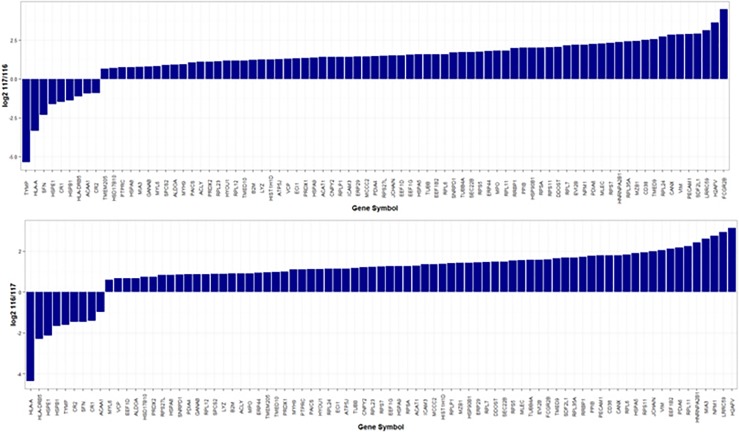
Proteins differentially expressed between MM-PC and ND-PC a. MM-PC labeled with iTRAQ reagent 117 and ND-PC labeled with iTRAQ reagent 116. b. MM-PC labeled with iTRAQ reagent 116 and ND-PC labeled with iTRAQ reagent 117. MM- PC = Multiple myeloma plasma cell; ND-PC = Normal donor plasma cell; iTRAQ = Isobaric tag for relative and absolute quantification.

**Figure 3:**
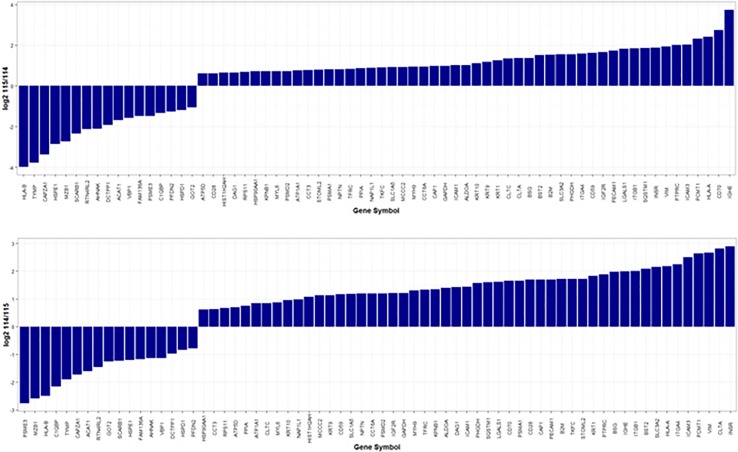
Proteins differentially expressed between U266 cell line and RPMI-8226 cell line a. U266 cell line labeled with iTRAQ reagent 115 and RPMI-8226 cell line labeled with iTRAQ reagent 114. b. U266 cell line labeled with iTRAQ reagent 114 and RPMI-8226 labeled with iTRAQ reagent 114. iTRAQ = Isobaric tag for relative and absolute quantification.

A total of 81 proteins were found differentially expressed between MM-PC and ND-PC, being 72 upregulated and nine downregulated (Figure 2), whereas U266 cell line vs. RPMI-8226 cell line presented 61 differentially expressed proteins, being 51 upregulated and 10 downregulated (Figure 3). For a detailed information about differentially expressed proteins (MM-PC vs. ND- PC and U266 vs. RPMI-8226), please see Supplemental Tables I and II.

**Figure 4:**
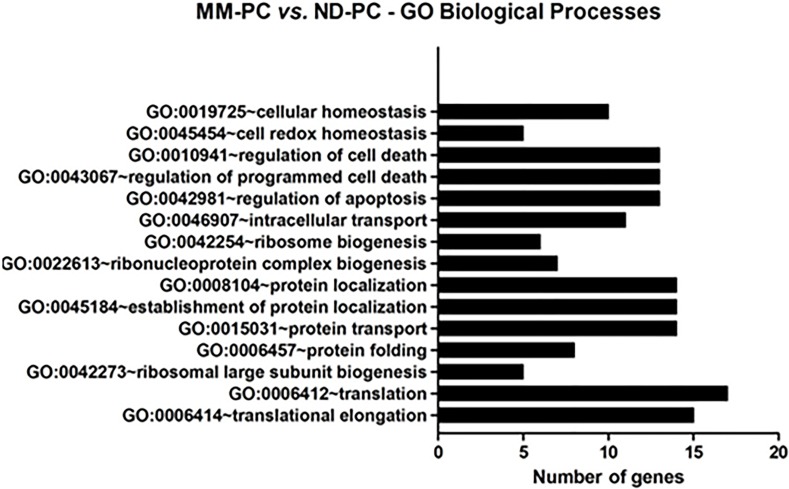
Most enriched GO biological processes terms between MM-PC and ND-PC, after bioinformatics analyses GO = Gene ontology; MM-PC = Multiple myeloma plasma cell; ND-PC = Normal donor plasma cell.

MM-PC vs. ND-PC comparisons: using DAVID Bioinformatics Resources 6.7 and adjusted p-value by Benjamini method (p<0.05), among our list of upregulated proteins, the most enriched Gene Ontology (GO)-biological processes terms were related to protein biosynthesis, cell death regulation and cell homeostasis (Figure 4), and the most enriched Kyoto Encyclopedia of Genes and Genomes (KEGG)-pathway was ribosome. With regard to protein biosynthesis, 15 out of 35 (43%) were ribosomal proteins, showing agreement between GO-Biological Processes and KEGG categories. Other protein biosynthesis functions differentially expressed between tumor and normal PC were related to translational elongation, protein folding, trafficking and localization. Among them, we highlight NPM1 and HSP70/HSP90 families.

On the other hand, when we analyzed our list of downregulated proteins, using the same cut-off criteria, there were no GO-biological processes terms or KEGG- pathways significantly enriched. However, the function of the most interesting downregulated proteins was manually annotated and checked in Entrez Gene database from NCBI [16]. We found some downregulated proteins in MM-PC that seem to play important role in cancer immune evasion mechanisms such as: HLA-A, HLA- DRB5, CR1, and CR2.

U266 vs. RPMI-8226 comparisons: using the same method described above, we found no GO-biological process terms significantly enriched among up- or downregulated proteins. However, among upregulated proteins in U266 cell line in comparison with RPMI-8226 cell line, the most enriched categories in KEGG-pathway were: cell adhesion molecules (CAMs) (ICAM1, ICAM3, PTPRC, PECAM1, HLA-A, ITGA4, ITGB1 and CD28) and viral myocarditis (also comprising ICAM1, HLA-A and CD28 plus DAG1, MYH9).

**Figure 5:**
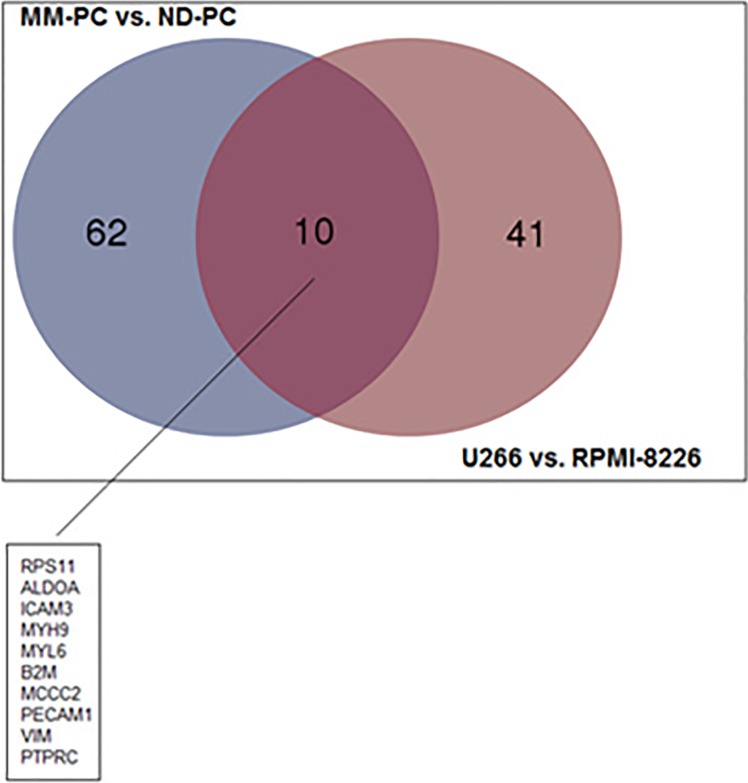
Venn Diagram representing the overlapping of upregulated proteins between both comparisons (MM-PC vs ND-PC and U266 vs RPMI-8226) MM-PC = Multiple myeloma plasma cell; ND-PC = Normal donor plasma cell.

**Figure 6:**
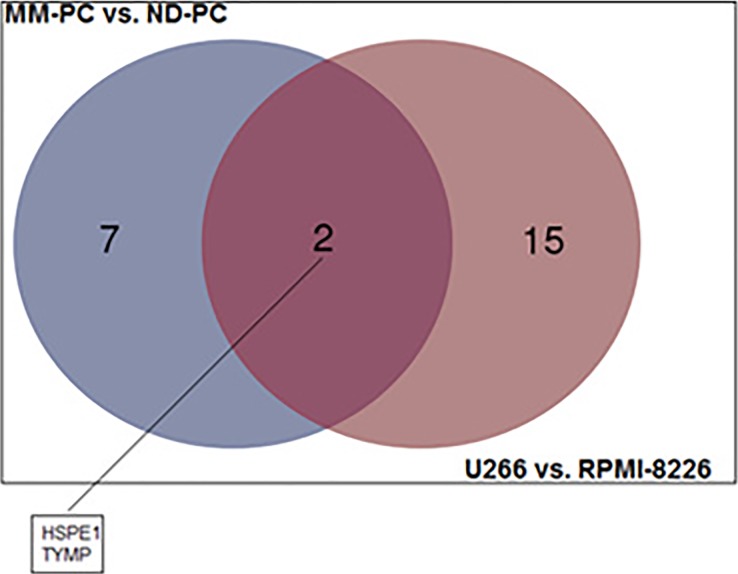
Venn diagram representing the overlapping of downregulated proteins between both comparisons (MM-PC vs ND-PC and U266 vs RPMI-8226) MM-PC = Multiple myeloma plasma cell; ND-PC = Normal donor plasma cell.

MM-PC vs. ND-PC and U266 vs. RPMI-8226 common features: among upregulated proteins, we found an overlap of 10 proteins between patient samples and cell lines (Figure 5), such as, adhesion molecules (ICAM3 and PECAM1), which can activate a number of signaling pathways promoting survival, proliferation, migration and drug resistance of MM-PC [17,18]; B2M, which is a well-known poor prognosis biomarker for MM patients; PTPRC, a protein tyrosine phosphatase receptor, which regulate a variety of cellular processes, including cell growth, differentiation, mitosis, and oncogenic transformation. Regarding downregulated proteins, we found an overlap of only two proteins (Figure 6).

## DISCUSSION

The present study highlighted the importance of protein biosynthesis machinery upregulation, such as NPM1 and HSP70/HSP90 families, as well as downregulation of some components of immune response, such as, MHC class I and II molecules, and complement receptors. Inhibition or enhancement of these proteins could be tested *in vitro*, in order to find more effective therapeutic strategies for MM treatment. The other contribution of the present study was to provide abundant information about MM-PC proteome in comparison with ND-PC, which can be further explored *in silico*, as well as *in vitro* assays, and also be validated in a larger number of patients. Moreover, we also provide comprehensive information about U266 cell line proteome (using the cell line RPMI-8226 as a control), and could confirm some primary tumor's findings.

After enrichment analyses of our results, protein biosynthesis proved to be one of the most significantly biological processes upregulated in MM-PC. Protein biosynthesis is an extremely important process for normal function of our cells and organisms as a whole and, therefore, is strictly regulated in order to ensure that it occurs properly [19]. The deregulation of protein biosynthesis is related to a number of events that contribute to the maintenance and progression of cancer, such as, cell proliferation, survival, and angiogenesis [19]. In line with our findings, previous studies demonstrated the upregulation of genes involved in protein biosynthesis, particularly ribosomal protein genes, when comparing MM-PC of patients with and without hyperdiploidy [20,21,22]. In MM-PC and ND-PC comparisons, among upregulated proteins involved in protein biosynthesis, NPM1 deserves attention, since its upregulation was also found in MM patients with hyperdiploidy [21,22] and Maggi et al. (2008) [23] demonstrated in other cell types that NPM1 upregulation enhances protein biosynthesis and the export of newly synthesized rRNAs. In addition, some other proteins involved in protein assembly and folding were also upregulated, such as CANX and PPIB.

In our study, we found upregulation of HSP70 members in MM-PC, including HSPA5, HSPA8 and HSPA9, and a member of HSP90 family - HSP90B1. Heat shock proteins (HSP) are members of chaperone families expressed by cells under normal and stress conditions. Their main physiological function is to maintain cellular protein homeostasis. For this purpose, they act in different levels, such as, proper folding of nascent polypeptides, misfolding protein degradation, intracellular trafficking, among others [24–28]. However, several studies have found HSP upregulation in a number of solid tumors and hematologic malignancies, favoring tumor proliferation, invasion and metastasis, as well as protecting tumor cells from apoptosis and from anti-tumor immune response [29]. Clinical studies using HSP90 inhibitors in cancer patients failed to confirm preclinical favorable results [30]. This can be explained because HSP90 inhibition causes a compensatory increase of HSP70 members [30]. Therefore, some groups had proposed the inhibition of HSP70 family members alone or in combination with HSP90 inhibitors, and they have achieved quite promising results [31,32,33]. The inhibition of HSPA8 (also known Hsc70) in MM has been evaluated and the results were very positive [33]. Our results suggest that the inhibition of other members of HSP70 family, as well as proteins with similar functions, should be tested *in vitro* and *in vivo*, in order to expand the range of therapeutic targets.

The concept of tumor immune surveillance was first proposed in the 70s [34] and it assumes that the immune system is capable of identifying and eliminating pre-cancerous and cancerous cells, before they can establish the disease [35]. However, since immunocompetent individuals can indeed develop cancer, a more appropriate concept is immunoediting, which is divided into three phases: elimination, equilibrium and escape [36]. In the latter, tumor cells develop a number of mechanisms to evade immune response and thereby enable the disease progression [37]. Among these arsenal, downregulation or complete loss of MHC class I expression have been described in several types of cancer [38], including MM [39]. In our study, despite upregulation of β2- microglubulin (light chain of MHC class I and a well- known poor prognosis biomarker in MM patients), HLA-A (heavy chain of MHC class I) was downregulated in MM-PC, suggesting a possible escape mechanism from cytotoxic T lymphocytes action by MM humans cells. In line with the results obtained in primary tumor samples, β2-microglubulin and HLA-B were, respectively, up- and downregulated in U266 cell line when compared to RPMI-8226. However, different than it was expected, HLA-A was upregulated in U266 cell line. This finding may be due to comparison of two tumor cell lines and, in this situation, MHC class I proteins expression maybe not a relevant differential criteria. In our study, we observed a downregulation of a molecule - HLA-DRB5 - that belongs to MHC class II, corroborating the idea of immune evasion.

Still in relation to the immune response, complement system plays a key role in innate immune response and, among its many functions, it play an important role against infections [40]. Since MM patients are more susceptible to infections, some studies have attempted to evaluate if the complement system is impaired in these patients [41,42]. It is believed that the complement system plays an important role in anti-tumor immunity [43] and, therefore, when its function is impaired, besides patient susceptibility to infection they are also more prone to develop some kinds of cancer. In our work, we found the complement receptors type 1 and type 2, CR1 and CR2, (also known as, CD21 and CD35, respectively) downregulated when MM-PC are compared to ND-PC. CD21/CD35 are very important for B cell activation and maturation [44]. Thus, CD21/CD35 downregulation on MM-PC might impair B cell maturation and activation, figuring a potential immune escape mechanism for MM pathogenesis or maintenance.

Regarding MM cell lines, after functional enrichment analyses, some adhesion molecules proved to be the most interesting proteins upregulated in U266 cell line in comparison with RPMI-8226 cell line. Among these molecules, some of them deserve greater attention, such as CD28, ITGB1 and ICAM1. Recently, Murray et al. (2014) [45] have proposed that CD28 molecule, through PI3K/Akt pathway, plays a key role in MM cells survival and resistance to chemotherapy. ITGB1, an intregrin, is very important for cell adhesion and recognition in a variety of processes. In MM, it exerts an important role in the interaction between MM-PC and bone marrow stromal cells, which is essential for MM progression. ICAM1 is a cell surface glycoprotein, however, it is typically expressed by bone marrow stromal cells and interacts with ITGB2 and MUC-1, both expressed by MM-PC. This interaction promotes activation of some pathways that contribute to MM-PC.

When we compared our lists of upregulated proteins in MM-PCs and U266 cell line, we found an overlap of some proteins that might contribute, directly or indirectly, to disease progression. However, when we compared our lists of downregulated proteins in MM-PCs and U266 cell line, the results were different from the expected, since HSPE1 is a heat shock protein that acts as a chaperonin and TYMP is a proangiogenic factor. For both, we expected increased expression in MM-PC but we found downregulation.

A clear limitation of our study was the absence of specificity of the kit used to extract membrane proteins. However, instead of a disadvantage, enrichment analyses enabled us to find many interesting proteins differentially expressed in other cellular compartments than plasma membrane. Other potential limitation is the fact that MM cases and controls were not matched by age. In our opinion, there is also no ideal control for MM-PC, since normal PC are in a limited number in peripheral blood (2/μL) [46] and BM of healthy people (<5%), and those obtained from people who have undergone tonsillectomy are derived from an inflammatory tissue and have a strong proliferative profile compared to normal bone marrow PC. However, we chose PC from palatine tonsils to ensure a sufficient amount of protein for mass spectrometry analyses, and since our results showed some well-known differentially expressed proteins in MM-PC, we can infer that gene expression of PC from palatine tonsils is, at least in part, similar to that of PC derived from BM of healthy individuals and, thus, it can figure as a normal control. Besides, another limitation of our study was the absence of mass spectrometry validation. Since MM is a rare disease and all samples collected were used for mass spectrometry analyses, we were not able to validate the most relevant proteins. However, our main goal was to provide comprehensive information about MM-PC proteome with some biological processes and pathways suggestions for further exploration.

In summary, our results showed that protein biosynthesis machinery is deregulated in MM-PC, mainly ribosomal proteins and proteins from HSP70 and HSP90 families, which are already being studied in MM as potential therapeutic targets. Besides, we also found downregulation of some proteins of immune system that are essential for antigen processing and presentation, as well as for complement system activation.

## MATERIAL AND METHODS

### Patients

Between February 2011 and June 2012, eight new cases of MM were enrolled at University Hospital São Paulo, São Paulo, Brazil. The diagnosis of MM was based on the International Myeloma Working Group [3] and information on the stage of the tumor was obtained according to Durie-Salmon criteria [47]and the International Staging System (ISS) [48]. Seven unmatched controls were included from a cohort of patients who underwent tonsillectomy at University Hospital São Paulo, São Paulo, Brazil. This study also relied on the use of two MM cell lines (U266, RPMI-8226), for comparative purposes.

### Bone Marrow and Palatine Tonsil Samples

After informed consent, BM aspirates were collected from MM cases at diagnosis, before any treatment (no chemotherapy, no corticosteroids, no bisphosphonates, no proteasome inhibitors, and no immunomodulatory drugs). Palatine tonsils from controls were collected at the moment of tonsillectomy, after informed consent signature according to the criteria described above.

### Sorting of Plasma Cells (PC)

Immediately after collection, BM aspirates from MM patients underwent separation of PC (MM-PC), using Magnetic Cell Sorting of Human Cells (MACS) methodology (MiltenyiBiotec, Bergisch Gladbach, Germany). The marker used for MM-PC sorting was CD138 antigen, which is expressed in normal and neoplastic PC surface, but neither on circulating B and T lymphocytes, nor monocytes. After surgical removal, palatine tonsils were immediately transferred to Petri dishes containing DPBS (1X), and submitted to small superficial cuts with a scalpel. Then, they were washed with DPBS (1X) in order to obtain a cell solution. Finally, cell solutions underwent PC separation by the same method as MM-PC. PC derived from palatine tonsils, i.e., from normal donors (ND-PC) and MM-PC were frozen in 45% of RPMI 1640 medium, 45% of fetal bovine serum (FBS), and 10% of DMSO, and stored in liquid nitrogen until use.

### Culture and Cytogenetic Analyses of MM Cell Lines

MM Cell lines were cultured in RPMI 1640 medium, supplemented with 10% of FBS, 1% of L-glutamine, 1% of MEM Nonessential Amino Acids, and 40 mg/mL of garamycin. The cultures were fed three times a week and incubated at 37°C in 5% CO.

MM cell lines (U266 and RPMI-8226) underwent FISH analyses, and the following markers were evaluated: deletion of chromosome 13 (13q14 LSI 13 [RB1] Spectrum Orange, Vysis) and deletion of 17p (17p13.1 LSI p53 Spectrum Orange, Vysis) [49].

### Protein Extraction and Quantification

Cell surface protein extraction and quantification were performed using, respectively, the Pierce Cell Surface Protein Isolation kit and the Pierce 660 nm Protein Assay (Thermo Scientific, Rockford, IL, USA), according to the manufacturer's instructions.

In order to obtain sufficient protein concentration for mass spectrometry analyses, human samples were combined in pools: MM-PC (n = 8) and ND-PC (n = 7).

### Steps Before Mass Spectrometry Analyses

Before mass spectrometry analyses, proteins underwent the following steps: trypsin digestion, desalting, labeling with 4-plex iTRAQ (Isobaric Tag for Relative and Absolute Quantification) kit (Applied Biosystems, Framingham, MA, USA), new desalting and detergent removal. Technical replicates of each sample (peptide solution) were labeled with a different isobaric tag. During labeling with iTRAQ, human samples were grouped in pools as described above, and compared as follows: MM-PC vs. ND-PC, and U266 cell line vs. RPMI-8226 cell line.

### Mass Spectrometry Analyses

Samples were analyzed in the LTQ Orbitrap Velos mass spectrometer, a very sensitive device, coupled with LC-MS/MS by EASY-nLC II system (liquid nanocromatography), and data were collected using the database Proteome Discoverer v.1.3 with Sequest, in comparison with the NCBI Database Human IPI v.3.86.

### Statistics and Bioinformatics Analyses

First, proteins that have not had sufficient expression to be detected in the technical replicate were excluded. Cut-off criteria to define differentially expressed proteins were arbitrarily established - fold-change of 1.5 for upregulation and 0.6 for downregulation - these values were chosen in order to identify even minimal differences in protein expression between groups. Functional enrichment analyses were performed separately for the upregulated and downregulated proteins, using the software DAVID [50].

### Ethical Aspects

This study was submitted and approved by the *UNIFESP* Ethics Committee, under the number of 1533/10.

## SUPPLEMENTARY MATERIAL TABLES



## References

[1] Solly S (1844). Remarks on the pathology of mollities ossium with cases. Med Chir Trans.

[2] Kyle RA, Rajkumar SV (2008). Multiple myeloma ASH 50th anniversary review multiple myeloma. Blood [Internet].

[3] Kyle RA, Rajkumar SV (2009). Criteria for diagnosis, staging, risk stratification and response assessment of multiple myeloma. Leukemia.

[4] Dimopoulos MA, Terpos E (2010). Multiple myeloma. Ann Oncol.

[5] American Cancer Society What are the key statistics about multiple myeloma? [Internet]. http://www.cancer.org/cancer/multiplemyeloma/detailedguide/multiple-myeloma-keystatistics.

[6] Howlader N, Noone AM, Krapcho M, Garshell J, Miller D, Altekruse SF, Kosary CL, Yu M, Ruhl J, Tatalovich Z, Mariotto A, Lewis DR, Chen HS, Feuer EJ, Cronin KA SEER Cancer Statistics Review, 1975-2012. http://seer.cancer.gov/csr/1975_2012/.

[7] Kumar SK, Rajkumar SV, Dispenzieri A, Lacy MQ, Hayman SR, Buadi FK, Zeldenrust SR, Dingli D, Russell SJ, Lust JA, Greipp PR, Kyle RA, Gertz MA (2008). Improved survival in multiple myeloma and the impact of novel therapies. Blood.

[8] Laubach JP, Richardson PG, Anderson KC (2010). The evolution and impact of therapy in multiple myeloma. Med Oncol.

[9] Mbeunkui F, Johann DJ (2009). Cancer and the tumor microenvironment: A review of an essential relationship. Cancer Chemother Pharmacol.

[10] Corn PG (2012). The tumor microenvironment in prostate cancer: Elucidating molecular pathways for therapy development. Cancer Manag Res.

[11] Zhou J, Mauerer K, Farina L, Gribben JG (2005). The role of the tumor microenvironment in hematological malignancies and implication for therapy. Front Biosci.

[12] Mitsiades CS, Mitsiades NS, Munshi NC, Richardson PG, Anderson KC (2006). The role of the bone microenvironment in the pathophysiology and therapeutic management of multiple myeloma: Interplay of growth factors, their receptors and stromal interactions. Eur J Cancer.

[13] Ge F, Tao S, Bi L, Zhang Z, Zhang XE (2011). Proteomics: addressing the challenges of multiple myeloma MM: the disease proteomics technologies.

[14] Cumova J, Potacova A, Zdrahal Z, Hajek R (2011). Proteomic analyses in multiple myeloma research. Mol Biotechnol.

[15] Lokhorst HM, Plesner T, Gimsing P (2013). Phase I/II dose- escalation study of Daratumumab in patients with relapsed or refractory multiple myeloma. J Clin Oncol.

[16] Maglott D (2004). Entrez Gene: gene-centered information at NCBI. Nucleic Acids Res.

[17] Katz BZ (2010). Adhesion molecules-The lifelines of multiple myeloma cells. Semin Cancer Biol.

[18] Neri P, Bahlis NJ (2012). Targeting of adhesion molecules as a therapeutic strategy in multiple myeloma. Curr Cancer Drug Targets.

[19] Bhat M, Robichaud N, Hulea L, Sonenberg N, Pelletier J, Topisirovic I (2015). Targeting the translation machinery in cancer. Nat Publ Gr.

[20] Agnelli L, Fabris S, Bicciato S, Basso D, Baldini L, Morabito F, Verdelli D, Todoerti K, Lambertenghi-Deliliers G, Lombardi L, Neri Al (2007). Upregulation of translational machinery and distinct genetic subgroups characterise hyperdiploidy in multiple myeloma. Br J Haematol.

[21] Chng WJ, Kumar S, Vanwier S, Ahmann G, Price-Troska T, Henderson K, Chung TH, Kim S, Mulligan G, Bryant B, Carpten J, Gertz M, Rajkumar SV (2007). Molecular dissection of hyperdiploid multiple myeloma by gene expression profiling. Cancer Res.

[22] Weinhold N, Moreaux J, Raab MS, Hose D, Hielscher T, Benner A, Meissner T, Ehrbrecht E, Brough M, Jauch A, Goldschmidt H, Klein B, Moos Ml (2010). NPM1 is overexpressed in hyperdiploid multiple myeloma due to a gain of chromosome 5 but is not delocalized to the cytoplasm. Genes, Chromosomes & Cancer.

[23] Maggi LB, Kuchenruether M, Dadey DY, Schwope RM, Grisendi S, Townsend RR, Pandolfi PP, Weber JD (2008). Nucleophosmin serves as a rate-limiting nuclear export chaperone for the Mammalian ribosome. Mol Cell Biol.

[24] Craig EA (1993). Chaperones: helpers along the pathways to protein folding. Science.

[25] Hartl FU (1996). Molecular chaperones in cellular protein folding. Nature.

[26] Hartl FU, Hayer-Hartl M (2002). Molecular chaperones in the cytosol: from nascent chain to folded protein. Science.

[27] Muralidharan S, Mandrekar P (2013). Cellular stress response and innate immune signaling: integrating pathways in host defense and inflammation. J Leukoc Biol.

[28] Udono H, Ichiyanagi T, Mizukami S, Imai T (2009). Heat shock proteins in antigen trafficking--implications on antigen presentation to T cells. Int J Hyperthermia.

[29] Zhang L, Fok JHL, Davies FE (2014). Heat shock proteins in multiple myeloma. Oncotarget.

[30] Trepel J (2010). Targeting the dynamic HSP90 complex in cancer. Nat. Rev. Cancer.

[31] Chatterjee M, Andrulis M, Stuhmer T, Muller E, Hofmann C, Steinbrunn T, Heimberger T, Schraud H, Kressmann S, Einsele H, Bargou RC (2012). The PI3K/Akt signaling pathway regulates the expression of Hsp70, which critically contributes to Hsp90-chaperone function and tumor cell survival in multiple myeloma. Haematologica.

[32] Davenport EL, Zeisig A, Aronson LI, Moore HE, Hockley S, Gonzalez D, Smith EM, Powers MV, Sharp SY, Workman P, Morgan GJ, Davies FE (2010). Targeting heat shock protein 72 enhances Hsp90 inhibitor-induced apoptosis in myeloma. Leukemia.

[33] Zhang L, Fok JJ, Mirabella F (2013). Hsp70 inhibition induces myeloma cell death via the intracellular accumulation of immunoglobulin and the generation of proteotoxic stress. Cancer Letters.

[34] Dunn GP, Bruce AT, Ikeda H, Old LJ, Schreiber RD (2002). Cancer immunoediting: from immunosurveillance to tumor escape. Nat Immunol.

[35] Swann JB, Smyth MJ (2007). Immune surveilance of tumors. J Clin Invest.

[36] Dunn GP, Old LJ, Schreiber RD (2004). The three Es of cancer immunoediting. Annu Rev Immunol.

[37] Hanahan D, Weinberg RA (2000). The hallmarks of cancer. Cell.

[38] Aptsiauri N, Cabrera T, Mendez R, Garcia-Lora A, Ruiz-Cabello F, Garrido F (2007). Role of altered expression of HLA class I molecules in cancer progression. Adv Exp Med Biol.

[39] Klippel ZK, Chou J, Towlerton AM, Voong LN, Robbins P, Bensinger WI, Warren EHl (2014). Immune escape from NY-ESO- 1-specific T cell therapy via loss of heterozygosity in the MHC. Gene Ther.

[40] Ricklin D, Lambris JD (2007). Complement-targeted therapeutics. Nat Biotechnol.

[41] Kraut EH, Sagone AL (1981). Alternative pathway of complement in multiple myeloma. Am J Hematol.

[42] Lugassy G, Platok I, Schlesinger M (1999). Hypocomplementemia in multiple myeloma. Leuk Lymphoma.

[43] Macor P, Tedesco F (2007). Complement as effector system in cancer immunotherapy. Immunol Lett.

[44] Carroll MC (1998). CD21/CD35 in B cell activation. Semin Immunol.

[45] Murray ME, Gavile CM, Nair JR, Koorella C, Carlson LM, Buac D, Utley A, Chesi M, Bergsagel PL, Boise LH, Lee KP (2014). CD28-mediated pro-survival signaling induces chemotherapeutic reistance in multiple myeloma. Blood.

[46] Caraux A, Klein B, Paiva B, Bret C, Schmitz A, Fuhler GM, Bos NA, Johnsen HE, Orfao A, Perez-Andres M (2010). Circulating human B and plasma cells. Age-associated changes in counts and detailed characterization of circulating normal CD138- and CD138+ plasma cells. Haematologica.

[47] Durie BG, Salmon SE (1975). A clinical staging system for multiple myeloma. Correlation of measured myeloma cell mass with presenting clinical features, response to treatment, and survival. Cancer.

[48] Greipp PR, Miguel JS, Dune BGM, Crowley JJ, Barlogie B, Bladé J, Boccadoro M, Child JA, Avet-Loiseau H, Kyle RA, Lahuerta JJ, Ludwing H, Morgan G (2005). International staging system for multiple myeloma. J Clin Oncol.

[49] Mikhael JR, Dingli D, Roy V, Reeder CB, Buadi FK, Hayman SR, Dispenzieri A, Fonseca R, Shert T, Kyle RA, Lin Y, Russel J, Kumar S (2013). Management of newly diagnosed symptomatic multiple myeloma: updated Mayo Stratification of Myeloma and Risk-Adapted Therapy (mSMART) consensus guidelines 2013. Mayo Clinic Proc.

[50] Huang DW, Sherman BT, Lempicki RA (2009). Systematic and integrative analyses of large gene lists using DAVID Bioinformatics Resources. Nature Protoc.

