# Supplementary material for: Multiple myeloma cell lines and primary tumors proteoma: protein biosynthesis and immune system as potential therapeutic targets
Source: Genes Cancer. 2015 Nov;6(11-12):462–71. doi: 10.18632/genesandcancer.88 (PMC4701225; doi:10.18632/genesandcancer.88)
Supplement: Supplementary file 1 [file ganc-06-462-s001.pdf]

Multiple myeloma cell lines and primary tumors proteoma: protein biosynthesis and immune system as potential therapeutic targets

Supplementary Material

Supplemental Table I: Complete list of genes encoding for the differentially expressed proteins in MM-PC using ND-PC as control.

Proteins upregulated in MM-PC (using ND-PC as controls)			
Official Symbol	Description	117/116	116/117
FCGR2B	Fc fragment of IgG, low affinity IIb, receptor (CD32)	22,493	3,031
H2AFV	H2A histone family, member V	12,406	8,775
LRRC59	leucine rich repeat containing 59	8,779	7,638
SDF2L1	stromal cell-derived factor 2-like 1	7,554	3,22
PECAM1	platelet/endothelial cell adhesion molecule 1	7,518	3,468
VIM	vimentin	7,337	4,131
CANX	calnexin	7,211	3,485
RPL24	ribosomal protein L24	6,698	2,201
TMED9	transmembrane emp24 protein transport domain containing 9	5,941	3,127
CD38	CD38 molecule	5,71	3,473
MZB1	marginal zone B and B1 cell-specific protein	5,469	2,705
RPL35A	ribosomal protein L35a	5,347	3,232
HNRNPA2B1	heterogeneous nuclear ribonucleoprotein A2/B1	5,199	5,402
RPS7	ribosomal protein S7	4,99	2,393
MLEC	malectin	4,825	2,939
PDIA6	protein disulfide isomerase family A, member 6	4,778	4,531
NPM1	nucleophosmin (nucleolar phosphoprotein B23, numatrin)	4,64	6,739
EVI2B	ecotropic viral integration site 2B	4,594	3
RPL7	ribosomal protein L7	4,443	2,757
DDOST	dolichyl-diphosphooligosaccharide--protein glycosyltransferase subunit (non-catalytic)	4,212	2,801
RPS11	ribosomal protein S11	4,118	3,839
RPSA	ribosomal protein SA	4,07	2,426
HSP90B1	heat shock protein 90kDa beta (Grp94), member 1	4,059	2,712
PPIB	peptidylprolyl isomerase B (cyclophilin B)	4,047	3,45
RRBP1	ribosome binding protein 1	4,016	3,291
RPL11	ribosomal protein L11	3,537	4,767
MPO	myeloperoxidase	3,533	1,89
ERP44	endoplasmic reticulum protein 44	3,495	1,923
RPS5	ribosomal protein S5	3,375	2,924
SEC22B	SEC22 vesicle trafficking protein homolog B (S. cerevisiae) (gene/pseudogene)	3,341	2,812
TUBB4A	tubulin, beta 4A class IVa	3,301	2,997

SNRPD1	small nuclear ribonucleoprotein D1 polypeptide 16kDa	3,262	1,806
RPL6	ribosomal protein L6	3,021	3,576
EEF1B2	eukaryotic translation elongation factor 1 beta 2	3,016	4,342
TUBB	tubulin, beta class I	3,013	2,279
HSPA5	heat shock 70kDa protein 5 (glucose-regulated protein, 78kDa)	2,988	3,758
EEF1G	eukaryotic translation elongation factor 1 gamma	2,959	2,415
EEF1D	eukaryotic translation elongation factor 1 delta (guanine nucleotide exchange protein)	2,866	1,593
JCHAIN	joining chain of multimeric IgA and IgM	2,849	3,983
RPS27L	ribosomal protein S27-like	2,808	1,778
PDIA4	protein disulfide isomerase family A, member 4	2,795	1,827
MCCC2	methylcrotonoyl-CoA carboxylase 2 (beta)	2,734	2,587
ERP29	endoplasmic reticulum protein 29	2,733	2,752
ICAM3	intercellular adhesion molecule 3	2,686	2,583
RPLP1	ribosomal protein, large, P1	2,674	2,676
CNPY2	canopy FGF signaling regulator 2	2,67	2,334
ACAT1	acetyl-CoA acetyltransferase 1	2,665	2,437
HSPA9	heat shock 70kDa protein 9 (mortalin)	2,588	2,426
PRDX1	peroxiredoxin 1	2,551	2,003
ECI1	enoyl-CoA delta isomerase 1	2,508	2,207
VCP	valosin containing protein	2,477	1,589
ATP5J	ATP synthase, H+ transporting, mitochondrial Fo complex, subunit F6	2,428	2,217
HIST1H1D	histone cluster 1, H1d	2,394	2,597
LYZ	lysozyme	2,394	1,859
B2M	beta-2-microglobulin	2,356	1,886
TMED10	transmembrane emp24-like trafficking protein 10	2,273	1,984
RPL12	ribosomal protein L12	2,267	1,838
HYOU1	hypoxia up-regulated 1	2,258	2,187
RPL23	ribosomal protein L23	2,203	2,356
PRDX2	peroxiredoxin 2	2,181	1,687
ACLY	ATP citrate lyase	2,157	1,887
PAICS	phosphoribosylaminoimidazole carboxylase, phosphoribosylaminoimidazole succinocarboxamide synthetase	2,08	2,182
MYH9	myosin, heavy chain 9, non-muscle	1,929	2,154
ALDOA	aldolase A, fructose-bisphosphate	1,9	1,606
SPCS2	signal peptidase complex subunit 2 homolog	1,867	1,855
MYL6	myosin, light chain 6, alkali, smooth muscle and non-muscle	1,768	1,528
GANAB	glucosidase, alpha; neutral AB	1,746	1,827
MIA3	melanoma inhibitory activity family, member 3	1,722	6,142
HSPA8	heat shock 70kDa protein 8	1,684	1,781
PTPRC	protein tyrosine phosphatase, receptor type, C	1,677	2,171
HSD17B10	hydroxysteroid (17-beta) dehydrogenase 10	1,631	1,676
TMEM205	transmembrane protein 205	1,576	1,942

Proteins downregulated in MM-PC (using ND-PC as controls)			
TYMP	thymidine phosphorylase	0,024	0,33
HLA-A	major histocompatibility complex, class I, A	0,099	0,049
SFN	stratifin	0,201	0,364
HSPE1	heat shock 10kDa protein 1	0,324	0,228
CR1	complement component (3b/4b) receptor 1 (Knops blood group)	0,357	0,378
HSPB1	heat shock 27kDa protein 1	0,385	0,317
HLA-DRB5	major histocompatibility complex, class II, DR beta 5	0,459	0,205
ACAA1	acetyl-CoA acyltransferase 1	0,521	0,509
CR2	complement component (3d/Epstein Barr virus) receptor 2	0,534	0,361

MM-PC = *Multiple myeloma plasma cells;* ND-PC = *Normal donor plasma cells.*

Supplemental Table II: Complete list of genes encoding for the differentially expressed proteins in U266 cell line using RPMI-8226 cell line as control.

Proteins upregulated in U266 MM cell line (using RPMI-8226 cell line as control)			
**Official Symbol**	**Description**	**115/114**	**114/115**
IGHE	immunoglobulin heavy constant epsilon	13,427	3,985
CD70	CD70 molecule	6,706	3,140
HLA-A	major histocompatibility complex, class I, A	5,357	4,522
PCMT1	protein-L-isoaspartate (D-aspartate) O-methyltransferase	5,025	6,206
ICAM3	intercellular adhesion molecule 3	4,058	5,647
PTPRC	protein tyrosine phosphatase, receptor type, C	4,052	3,701
VIM	vimentin	3,823	6,325
INSR	insulin receptor	3,666	7,462
SQSTM1	sequestosome 1	3,633	3,043
ITGB1	integrin, beta 1 (fibronectin receptor, beta polypeptide, antigen CD29 includes MDF2, MSK12)	3,603	3,999
LGALS1	lectin, galactoside-binding, soluble, 1	3,528	3,063
PECAM1	platelet/endothelial cell adhesion molecule 1	3,312	3,248
IGF2R	insulin-like growth factor 2 receptor	3,181	2,314
CD59	CD59 molecule, complement regulatory protein	3,079	2,255
ITGA4	integrin, alpha 4 (antigen CD49D, alpha 4 subunit of VLA-4 receptor)	2,996	4,731
PHGDH	phosphoglycerate dehydrogenase	2,926	2,967
SLC3A2	solute carrier family 3 (amino acid transporter heavy chain), member 2	2,915	4,430
B2M	beta-2-microglobulin	2,895	3,283
BST2	bone marrow stromal cell antigen 2	2,853	4,228
BSG	basigin (Ok blood group)	2,592	3,950
CLTA	clathrin, light chain A	2,574	6,991
CLTC	clathrin, heavy chain (Hc)	2,545	1,798
KRT1	keratin 1, type II	2,383	3,569
KRT9	keratin 9, type I	2,271	2,191
KRT10	keratin 10, type I	2,159	1,935
ALDOA	aldolase A, fructose-bisphosphate	2,028	2,645
ICAM1	intercellular adhesion molecule 1	2,018	2,723
GAPDH	glyceraldehyde-3-phosphate dehydrogenase	1,988	2,319
CAP1	CAP, adenylate cyclase-associated protein 1 (yeast)	1,964	3,229
CCT6A	chaperonin containing TCP1, subunit 6A (zeta 1)	1,918	2,293
MYH9	myosin, heavy chain 9, non-muscle	1,917	2,476
MCCC2	methylcrotonoyl-CoA carboxylase 2 (beta)	1,900	2,182
SLC1A5	solute carrier family 1 (neutral amino acid transporter), member 5	1,896	2,279
TKFC	triokinase/FMN cyclase	1,877	3,289
NAP1L1	nucleosome assembly protein 1-like 1	1,854	1,972
PPIA	peptidylprolyl isomerase A (cyclophilin A)	1,840	1,689
TFRC	transferrin receptor	1,777	2,523
NPTN	neuroplastin	1,762	2,286
PSMA1	proteasome (prosome, macropain) subunit, alpha type, 1	1,753	3,154
STOML2	stomatin (EPB72)-like 2	1,734	3,289

CCT3	chaperonin containing TCP1, subunit 3 (gamma)	1,718	1,550
ATP1A1	ATPase, Na+/K+ transporting, alpha 1 polypeptide	1,701	1,795
PSMD2	proteasome (prosome, macropain) 26S subunit, non-ATPase, 2	1,655	2,300
MYL6	myosin, light chain 6, alkali, smooth muscle and non-muscle	1,655	1,836
KPNB1	karyopherin (importin) beta 1	1,651	2,532
HSP90AA1	heat shock protein 90kDa alpha (cytosolic), class A member 1	1,646	1,529
RPS11	ribosomal protein S11	1,616	1,585
DAG1	dystroglycan 1 (dystrophin-associated glycoprotein 1)	1,573	2,676
HIST1H2AH	histone cluster 1, H2ah	1,566	2,107
CD28	CD28 molecule	1,537	3,222
ATP5D	ATP synthase, H+ transporting, mitochondrial F1 complex, delta subunit	1,524	1,615
**Proteins downregulated in U266 MM cell line (using RPMI-8226 as control)**			
HLA-B	major histocompatibility complex, class I, B	0,064	0,178
TYMP	thymidine phosphorylase	0,073	0,268
CAPZA1	capping protein (actin filament) muscle Z-line, alpha 1	0,097	0,303
HSPE1	heat shock 10kDa protein 1	0,138	0,437
MZB1	marginal zone B and B1 cell-specific protein	0,150	0,166
SCARB1	scavenger receptor class B, member 1	0,197	0,427
RTN4RL2	reticulon 4 receptor-like 2	0,228	0,365
AHNAK	AHNAK nucleoprotein	0,234	0,456
DCTPP1	dCTP pyrophosphatase 1	0,265	0,511
ACAT1	acetyl-CoA acetyltransferase 1	0,313	0,330
VBP1	von Hippel-Lindau binding protein 1	0,338	0,457
FAM136A	family with sequence similarity 136, member A	0,357	0,444
PSME3	proteasome (prosome, macropain) activator subunit 3 (PA28 gamma; Ki)	0,360	0,148
C1QBP	complement component 1, q subcomponent binding protein	0,395	0,225
PFDN2	prefoldin subunit 2	0,417	0,578
HSPD1	heat shock 60kDa protein 1 (chaperonin)	0,442	0,560
GOT2	glutamic-oxaloacetic transaminase 2, mitochondrial	0,481	0,419
